# Medical and Physical Intelligence

**Published:** 1821-09

**Authors:** 


### Medical and Physical Intelligence.

THE *Phrenological Society of Edinburgh*, instituted at the commencement of the last year, appears, from the report of its Transactions just published, to be carrying into execution, with much spirit, the objects of its establishment. The Editor of this Journal is not a sectary of this system of Psychology;—he believes, as he intimated on a former occasion,* that the evidence hitherto adduced in its favour is not sufficient to prove its truth, whilst he considers that no arguments have hitherto been adduced by which it is absolutely refuted;†—but he nevertheless thinks that much benefit, especially to comparative anatomy and physiology, may be expected to result from this Society. Still more advantage would, probably, have resulted from it, had its fundamental laws been somewhat different. "The existence of this Society," it is said, "implies a Belief in the members, that the Brain is the organ of the mind, and that particular parts of it are the organs of particular mental faculties; and that these facts afford a key to the true philosophy of man." It is also stated, that the Society "hold the principles of Phrenology as no longer subject to doubt; and, while they recommend an attention to the subject to every reflecting and virtuous individual who considers a knowledge of himself and of human nature as an object of importance, they conceive themselves called upon to direct their own efforts rather to enlarge and apply the truths which phrenology reveals, than to strengthen the evidence on which the general principles rest." Such laws must exclude from the Society men who are qualified to promote its objects in an eminent degree. There are not a few physiologists who think that there may be some truth in this doctrine of phrenology, and who, while they entertain such an opinion, would readily contribute such observations as may occur to them, that tend either to prove or to illustrate its principles; but who will not enrol themselves amongst those who profess such a degree of belief in it as is indicated in the laws just transcribed.

It would appear, from the titles of the papers which have been read to the Society, that some very interesting discussions have taken place at their sessions; and a series of casts has been presented to the Society by Sir GEORGE MACKENZIE, comprising models of skulls of human beings, in the words of the Report, "from the lowest to the

* When noticing Mr. COMBE's Treatise on the subject, in the *Proëmium* to the forty-third volume.

† These remarks, it should, however, be understood, are made in regard to the general tenor of what Dr. GALL has advanced on the subject, and not to the psychological notions peculiar to Dr. SPURZHEIM; especially those, generally, which he has exprested in his *Essai Philosophique sur la Nature Morale et Intellectuelle de l'Homme.*

highest state of civilization."* The number of ordinary members, at the time of the publication of this report, was forty-two, amongst whom there are nine medical men. We should like to give a list of the whole, but our limits forbid it: we shall, therefore, only mention the names of the honorary members, who are Drs. Gall and Spurzheim, and Dr. Herberski, Professor of Medicine in the University of Wilna; and those of the "office-bearers," which are as follows:—George Combe, President; Sir G. S. Mackenzie, Bart. James Brownlee, and William Ritchie, Vice-Presidents; and Alexander Fleming, W. S. Secretary.

---

So much interest has been felt by the profession generally respecting the decision of the Select Committee of the House of Commons appointed to inquire into the establishment of the Ophthalmic Hospital in the Regent's Park, and the claims of Sir William Adams upon the public, as to make us think it adviseable to present our readers with the essential parts of the Report of that Committee. Mr. Keats, the late surgeon-general, first, in the year 1808, urged to the commander-in-chief of the army the necessity of some measures for the prevention and treatment of the ophthalmia which had lately become so prevalent amongst our soldiers, and which had already deprived upwards of one thousand of them of sight. On the 28th of October, 1809, Dr. Vetch, then staff-surgeon of the ophthalmia depôt at Bognor, (of whose knowledge and skill in the treatment of this complaint the Committee are induced to think very highly, from the evidence which they received in the course of this investigation,) addressed a letter to Mr. Knight, then inspector-general of hospitals, to the same intent.

"In the beginning of the year 1810, a Board was appointed, consisting of the three principal officers of the Army Medical Board and of eight of the most distinguished civil practitioners in London; and their report, which contained some suggestions as to the most effectual means of preventing the disorder from spreading by communication from infected persons, and as to the best mode of treating it in its early and acute stages, was circulated in general orders, for the guidance and information of the medical officers of the army.

"It does not appear, however, that this report contained any instructions as to the mode of treating the chronic or third stage of the ophthalmia: nor did it direct the attention of those, for whose guidance it was prepared, to the existence or treatment of those effects which the acute inflammation, in the earlier stages of the disorder, generally produces upon the membrane that lines the inner surface of the eye-lid.

"In the beginning of 1810, and afterwards in 1812, Sir W. Adams tendered to the military departments information as to the chronic or

---

* Copies of them, it is said, are sold to the public by O'Neil, of Cannongate, Edinburgh.

third stage of the disorder, and as to the mode of treating that stage, which he considered to be important, and at that time not generally known. In consequence of these communications from him, several out-pensioners of Chelsea Hospital, afflicted with blindness, were at different times placed under his care, as subjects for experiment as to the importance and efficacy of the practice which he recommended; and these communications and experiments ultimately led to the establishment of the Ophthalmic Hospital, at the head of which Sir Wm. Adams was placed.

"The objects of this institution were—

"First. To diffuse, generally, among the surgeons of the army, the knowledge of the best modes of treating the chronic and third stage of the disorder.

"Secondly. To diminish, if possible, the charge of the out-pensioners of Chelsea Hospital, by curing or relieving men who had received pensions for defective sight.

"Thirdly. To check, in some degree, the annual augmentation of the pension list, by treating men about to be discharged for defective sight, and by thus diminishing their claim to pension, as far as it might be founded upon the impaired state of their vision.

"With respect to the first point, the Committee have the satisfaction to find that this, which was the most important object, has been greatly promoted. The ophthalmia having, upon the return of our troops from Egypt, become, comparatively speaking, a new disease in this country, its proper treatment was at first imperfectly understood. It appears, however, that the attention of the medical department of the army has of late years been most successfully directed to this subject, and that the best modes of treating all the different stages of the ophthalmia are now well understood and practised in the army; and the Committee are satisfied that the establishment of the hospital, under Sir William Adams, has greatly contributed to promote this desirable object, not only by the direct opportunity it afforded of studying the various modes of practice, but indirectly, by the manner in which it appears to have excited the emulation and attention of other practitioners.

"With respect to the second point, indeed, it has been stated, that valid doubts were suggested how far it was in the power of the commissioners of Chelsea Hospital to take away, or diminish, any pension which they had granted under the provisions of the Act of the 46 Geo. III.: and, consequently, the Committee have not thought it necessary to direct their inquiries to this point, as no diminution of pensions already granted could, under any circumstances, have been effected.

"With respect to the third point, as but a few men so circumstanced have been placed in the hospital, it does not appear to the Committee that Sir William Adams has had sufficient opportunity of showing how far he could have effected this object, upon the scale originally proposed. But the general diffusion of knowledge among the medical officers of the army must necessarily lead to the accomplishment of this end.

"With regard to the future continuance of this establishment, it has been stated to the Committee by the department with which it originated, that, the main objects for which it was instituted having thus been attained, it does not appear that any public inconvenience would now arise from its discontinuance. In this opinion the Committee are disposed to concur; and they therefore recommend, that the establishment should be discontinued as soon as the proper arrangements can conveniently be effected.

"Upon the claims of Sir William Adams upon the public, the Committee report, that he has rested those claims upon two grounds.

"First. Upon his having been the means of promulgating to the army, and to the public, certain information as to the third or chronic stage of the ophthalmia, and its consequences: namely, that it is the general, if not invariable, effect of the inflammation in the acute stage of the disorder to produce, in a greater or less degree, what are termed granulations on the inner surface of the eye-lid; that these granulations render the patient subject to relapses, and are frequently the cause of blindness; that, during the relapses so happening, the patient is liable to become again infectious; and, therefore, that these granulations must invariably be looked for, and removed, before the patient can be effectually cured.

"Secondly. Upon his having attended the Ophthalmic Hospital since its first formation in 1817, without having hitherto received any remuneration for that duty.

"Upon the first point the Committee report, that the existence of these granulations, and the necessity of removing them, seems to have been known in very early times, and are adverted to in the works of Celsus, in the first century; of Paulus of Ægina, in the seventh; of Rhases the Arabian, in the tenth; and in the work of Sir William Reid, in the reign of Queen Anne.* That, consequently, no person in the present day can claim more than the merit of having revived knowledge which had fallen into neglect. The Committee do not feel it necessary to pronounce between the conflicting claims upon this head, or, by recommending a parliamentary reward for such revival, to decide to whom the merit properly belongs: they conceive that question is best left to the decision of the profession and of the public. But they are of opinion that Sir William Adams has, among others, been greatly instrumental in promulgating this knowledge, and in rendering it generally available.

"Upon the second point the Committee report, that, since the first establishment of the hospital in 1817, Sir William Adams has devoted to the duties arising out of his appointment, a large portion of that time which to a professional man is the source of income; and that, inasmuch as the time which he could apply to his private practice has thereby been much curtailed, his professional emoluments must also have been proportionally lessened. That he has performed the difficult duties of his appointment with the greatest zeal and assiduity;

* See the Review of Dr. VETCH's work, in a late Number of this Journal.

and that the Committee have been led to form the highest opinion of his skill and abilities as an oculist.

"The Committee, taking into consideration all the circumstances of the case, are induced to recommend that the sum of 4000*l.* should be paid to Sir William Adams, as a reward for the services which he has rendered to the public."

---

A PLANT, appertaining to a new genus, bearing a flower of extraordinary and prodigious dimensions, has lately been discovered in Sumatra, by the late Dr. ARNOLD, who accompanied the governor, Sir STAMFORD RAFFLES. It was found in a jungle, growing close to the ground under the bushes. It measured "a full yard across."—"The whole flower was of a very thick substance," Dr. Arnold says, "the petals and nectary being in but few places less than a quarter of an inch thick, and in some places three-quarters of an inch: the substance of it was very succulent. The calyx consisted of several roundish, dark-brown, concave leaves, which seemed to be indefinite in number, and were unequal in size. There were five petals attached to the nectary, which were thick, and covered with protuberances of a yellow-white, varying in size, the interstices being of a brick-red colour. The nectarium was cyathiform, becoming narrower towards the top. The centre of the nectarium gave rise to a large pistil, at the top of which were about twenty processes, somewhat curved and sharp at the end, resembling a cow's horns; there were as many smaller very short processes. A little more than half-way down, a brown cord, about the size of common whip-cord, but quite smooth, surrounded what perhaps is the germen; and a little below it was another cord, somewhat moniliform.—The petals were sub-rotund, twelve inches from the base to the apex, and the distance of the insertion of one petal to that of the opposite one was about a foot. The nectarium would hold twelve pints, and the weight of the flower was calculated to be about fifteen pounds.—I am not certain of the part I ought to call the stamina. If the moniliform cord surrounding the base of the pistil were sessile anthers, it must be a polyandrous plant; but I am uncertain what the large germen contained: perhaps, there might be concealed anthers within it. There were no leaves or branches to this plant; so that it is probable that the stems bearing leaves issue forth at a different period of the year. The soil where it grew was very rich, and covered with the excrement of elephants. A guide from the interior of the country said that such flowers were rare, but that he had seen several, and that the natives called them *krûbût*."

Mr. BROWN, on examining some of the buds, which have been presented to the Linnean Society, has found the anthers, but no part which could be regarded as a perfect pistil, or as indicating the probable nature, or even the exact place, of the ovary. The remains of the expanded flower exhibited the same structure; and another bud, also examined, proved likewise to be male.

Mr. Brown has connected his account of this plant, published in the thirteenth volume of the Linnean Transactions, with some considerations on certain points of the economy of the plant and on its affinities, that will be read with much interest by phytologists.

Since the first account of this plant was read to the Linnean Society, Sir Stamford Raffles has found that the krûbût is much more general and more extensively known than had been supposed. It seems to spring from the horizontal roots of those immense climbers which are attached like cables to the largest trees in the forest. The leaves have not yet been met with; neither has the fruit. It is said to be a many-seeded berry, the seeds being found in connexion with the processes on the summit of the pistil. Three months elapse between the first appearance of the bud and the full expansion of the flower, and the flower appears but once a year, at the conclusion of the rainy season.

Mr. Jack has ascertained several additional particulars respecting this flower. He has examined numerous specimens, sent from various parts of the country, which have proved that it is a parasite, and has a female as well as a male flower. It is parasitic on the roots and stems, of a ligneous species of *lissus*, which is the *lissus angustifolia* of Roxburgh. It appears to take its origin in some crack or hollow of the stem, and soon shows itself in the form of a round knob, which, when cut through, exhibits the infant flower enveloped in numerous bracteal sheaths, which successively open and wither away as the flower enlarges, until, at the time of full expansion, there are but very few remaining, which have somewhat of the appearance of a broken calyx. The flowers, as already stated, are unisexual, and consequently diœceous. The female differs but little in appearance from the male, but totally wants the globular anthers which are disposed round the lower side of the rim or margin of the central column of the male. In the centre of this column, in the female, are perceived several fissures traversing its substance, without order or regularity, and their surfaces are covered with innumerable minute seeds. The flower rots away not long after expansion, and the seeds are mixed with the pulpy mass. The male and female flowers can be distinguished by a section, not only when mature, but at every stage of their progress.

---

*On Phosphorescence.*—The phenomena of phosphorescence produced by exposure of bodies to light, have been very attentively observed lately by Mr. Heinrich, of Ratisbonne, who has made some new and interesting observations on them. The precautions taken by the observer were to remain, previous to the observation, thirty or forty minutes in a perfectly dark place; to expose the substances, the powers of which were to be observed, for not more than ten seconds to the light of a clear day; to keep them out of the rays of the sun, lest they should become heated; and to observe them in the same dark chamber to which he had previously retired. The observations were made in two different seasons, the summer and the winter. The following are the general results, but the account is very much compressed from Mr. Heinrich's paper.

Among natural bodies, some are phosphorescent, some not phosphorescent. Among the most phosphorescent are some diamonds, but not all, though no apparent difference in their external appearances could be perceived; some remained luminous from five seconds to one hour. The different effects of the coloured rays were remarkable: a good diamond exposed in the blue rays acquired a double phosphorescence, but did not become luminous at all in the red rays. All the fluor spars were highly phosphorescent, and also all the carbonates of lime.

Mr. Heinrich observes, that the phosphorescence of calcareous combinations varies with the acid in combination. Thus, the fluates were very phosphorescent, some of them remaining luminous for one hour. The carbonates follow: they are distinguished by the clear and white light they emit, which is such in some specimens as to enable a person to read by it; but it does not continue above thirty or forty-five seconds. The sulphates shine for a short time, but very faintly: the phosphates are still less favourable for the phenomena of phosphorescence by irradiation.

The calcareous combinations are succeeded by heavy spar or sulphate, and carbonate of baryta. Pure siliceous, aluminous, and magnesian earths, are not phosphorescent, though many of their native combinations are feebly so.

With regard to saline minerals, Mr. Heinrich considers their phosphorescent powers to be determined by the acid and base in combination. With the exception of amber and the diamond, no inflammable fossil becomes phosphorescent by irradiation. None of the metals are phosphorescent; metallic salts are moderately, and metallic oxides feebly, phosphorescent.

Vegetable substances are but bad phosphori. The wood of hot countries is better than that of our climate: the white hazel-tree shines brightly; an old sugar-cane, dates, and the inner part of the cocoa-nut, become finely phosphorescent. Cotton is very bad; dried plants are in general very feebly luminous; vegetable substances bleached are infinitely superior to the same substance not bleached: this is particularly observable in linen, paper, &c. Animal substances, containing carbonate of lime, as egg-shells, shells, corals, &c. are more phosphorescent than those containing phosphate of lime.

After the facts of which the above are the results, follow various interesting observations: thus, it is remarked that the duration of the light differs very much; the diamond and fluor shine for above an hour, no other fossil for more than a minute. Vividness and duration are in no relation to each other. With the exception of the diamond, the light of fossils is always white, whether they be illuminated by coloured rays or not. Sun-light is more effectual in producing the phenomena than day-light, but too long an exposure is disadvantageous. White bodies act more powerfully than coloured, and light coloured more than dark coloured. Bodies that are shining continue to shine when immersed in water. Difference of temperature has but little effect: ice is phosphorescent; at the same time it is observed that heat augments the intensity and diminishes the duration of the phosphorescence; cold has a contrary effect. Pure water and transparent fluids do not shine. The light appears to penetrate the substances considerably; for, when shining, if a groove be cut a line deep in the substance, it will be as luminous at the bottom of it as at the surface: finally, polishing injures, and in some cases destroys, the phosphorescent power.

Mr. Heinrich then speaks of artificial phosphori, and describes the process for preparing the Bolognian compound, and those of Canton, Baldwin, &c. Among animal and vegetable substances, many, not luminous at first, become so by being cooled or heated: some of these are the flesh of birds, dried tendons, burnt bones and horns, yolk of eggs, toasted cheese, roasted coffee, chestnuts, pease, &c.

Mr. Heinrich considers these phenomena of phosphorescence to be occasioned by the mere restitution or emission of the light absorbed by the phosphori during exposure to external sources. Among those which are most difficult to explain, are the preservation of the light by enveloping the luminous body perfectly, and its increased emission by the application of heat. If a diamond, the fluor spar of Siberia, or chlorophane, be exposed to light for some minutes, and be then covered with black wax, ink, or any substance, which will perfectly exclude air and light; on removing the envelope after some days, the body will still be found emitting light. This fact was known to Kircher and Beccaria. The second fact is the following:—when fluor spar which has been exposed to light has ceased to become luminous, it may be made to emit light by merely warming it with the breath or the hand. This effect may be obtained many times successively after only one irradiation, especially if at each time the warmth be a little increased: at last, warming ceases to produce the effect, but the simple exposure of the spar to the sun enables it anew to present the same appearances as before.—(*Bibl. Univ.* xv. p. 247.)

---

*Apple Bread.*—Mr. Duduit de Maizieres, a French officer of the king's household, has invented, and practised with great success, a method of making bread with common apples very far superior to potato bread. After having boiled one-third of peeled apples, he bruised them while quite warm into two-thirds of flour, including the proper quantity of yeast, and kneaded the whole without water, the juice of the fruit being quite sufficient. When this mixture had acquired the consistency of paste, he put it into a vessel, in which he allowed it to rise for about twelve hours. By this process he obtained a very excellent bread, full of eyes, and extremely palatable and light.—*New Monthly Mag.*

*Decomposition of Blood.*—Mr. Vauquelin had occasion to observe the changes produced in five years in the fluid obtained by washing coagulated bullocks' blood. It appears at first to have contained the serum and a considerable portion of the colouring matter of the blood, and the results at the end of the time mentioned were:

1. A large quantity of carbonic acid.
2. A large quantity of sulphuretted hydrogen.
3. A large quantity of acetic acid.
4. Ammonia which saturated these acids.
5. An acid and very fetid volatile oil, saturating part of the ammonia. These substances did not exist previously in the blood.
6. It appears that the fixed fatty matter which was found had existed in the blood previously, as a similar matter was found in recent blood.
7. That the albumen was almost entirely decomposed, slight traces only remaining, and so altered in its nature that it rather resembled glue than albumen.
8. That the colouring matter remained entirely unchanged.
9. That the blood did not appear to contain phosphorus.

Mr. Vauquelin remarks, that the quantity of sulphur in blood is much larger than is generally imagined, amounting to two grammes (about 30 grains) in a litre ($2\frac{1}{8}$ pints). This sulphur had separated spontaneously from the fluid, and formed a ring just above its surface on the glass.—*Ann. de Chim.* xvi. p. 363.

### METEOROLOGICAL JOURNAL.

By Messrs. William Harris and Co. 50, Holborn, London.

*From July* 20 *to August* 19, *inclusive.*

| Day of Month. | Moon. | Rain gauge | THERM. | | | BAROM. | | DeLuc's Hygro. | | WIND. | | ATMOSPHERIC VARIATION. | | |
|---|---|---|---|---|---|---|---|---|---|---|---|---|---|---|
| July | | | | | | | | | | | | | | |
| 20 | | | 66 | 68 | 63 | 29·87 | 29·82 | 57 | 57 | WSW | SW | Cloudy | Fine | |
| 21 | | ·09 | 67 | 69 | 60 | 29·80 | 29·80 | 57 | 56 | WSW | WSW | Fine | Rain | Fine |
| 22 | ☽ | ·08 | 63 | 68 | 57 | 29·61 | 29·61 | 59 | 56 | WSW | W | Showery | | Fine |
| 23 | | ·10 | 60 | 65 | 59 | 29·62 | 29·71 | 55 | 57 | W | SW | Showery | | |
| 24 | | ·19 | 61 | 66 | 58 | 29·76 | 29·63 | 57 | 57 | WSW | SW | Showery | | |
| 25 | | ·16 | 63 | 69 | 59 | 29·71 | 29·91 | 58 | 57 | WSW | W | Showery | | |
| 26 | | ·12 | 63 | 68 | 59 | 29·94 | 29·97 | 57 | 56 | WSW | W | Showery | | Fine |
| 27 | | | 63 | 67 | 58 | 29·96 | 30·03 | 55 | 56 | W | NW | Fine | | |
| 28 | | | 60 | 63 | 58 | 30·00 | 29·97 | 56 | 55 | ESE | WNW | Cloudy | | |
| 29 | ● | ·08 | 61 | 64 | 58 | 30·03 | 30·02 | 55 | 57 | W | W | Fine | | Rain |
| 30 | | ·19 | 59 | 63 | 60 | 29·93 | 29·89 | 61 | 68 | WSW | W | Rain | | Cloud. |
| 31 | | ·03 | 63 | 69 | 63 | 29·95 | 30·00 | 68 | 63 | WNW | WNW | Cloudy | | |
| Aug | | | | | | | | | | | | | | |
| 1 | | | 65 | 69 | 60 | 29·97 | 29·96 | 63 | 62 | W | W | Fine | | Cloud. |
| 2 | | ·11 | 63 | 68 | 63 | 30·11 | 30·11 | 61 | 60 | W | W | Fine | Rain | Cloud. |
| 3 | | | 66 | 71 | 62 | 30·11 | 30·11 | 61 | 60 | NNW | SSW | Fine | | |
| 4 | | | 67 | 72 | 63 | 30·00 | 30·05 | 57 | 55 | S | SE | Fine | | |
| 5 | | | 67 | 74 | 65 | 29·94 | 29·93 | 55 | 56 | S | SE | Fine | | |
| 6 | ☾ | | 70 | 75 | 60 | 29·86 | 29·96 | 58 | 57 | W | W | Fine | | |
| 7 | | ·05 | 65 | 69 | 58 | 30·01 | 30·02 | 57 | 57 | WNW | WNW | Fine | Sho'ry | Fine |
| 8 | | ·30 | 60 | 62 | 60 | 29·86 | 29·61 | 58 | 62 | WSW | W | Rain | | |
| 9 | | ·16 | 62 | 67 | 57 | 29·65 | 29·55 | 62 | 57 | WSW | W | Rain | Cloud. | Fine |
| 10 | | | 59 | 66 | 57 | 29·52 | 29·53 | 56 | 54 | W | W | Cloudy | Fine | |
| 11 | | ·20 | 62 | 68 | 50 | 29·60 | 29·75 | 57 | 55 | W | WNW | Cloudy | Rain | Cloud. |
| 12 | | | 61 | 68 | 60 | 29·90 | 29·97 | 53 | 56 | NW | WNW | Cloudy | | Fine |
| 13 | ○ | | 64 | 69 | 60 | 30·00 | 29·91 | 55 | 56 | WNW | W | Cloudy | Fine | Cloud. |
| 14 | | ·23 | 60 | 65 | 56 | 29·80 | 29·79 | 60 | 59 | WSW | N | Rain | | Cloud. |
| 15 | | | 59 | 69 | 60 | 29·94 | 30·02 | 59 | 59 | N | WNW | Fine | | |
| 16 | | | 65 | 71 | 64 | 30·07 | 30·08 | 60 | 60 | W | WSW | Cloudy | Fine | Cloud. |
| 17 | | | 67 | 74 | 64 | 30·14 | 30·04 | 61 | 66 | W | W | Cloudy | Fine | Cloud. |
| 18 | | | 66 | 69 | 57 | 30·06 | 30·14 | 60 | 60 | NW | NW | Cloudy | | Fog |
| 19 | | | 61 | 70 | 60 | 30·18 | 30·20 | 57 | 55 | SW | SSW | Fine | | |

The quantity of rain fallen in the month of July, is 2 inches and 25-100ths.

